# Assessment of anatomic variations of the brachial artery bifurcation using vascular Doppler ultrasound: a cross-sectional study

**DOI:** 10.1590/1677-5449.202301172

**Published:** 2024-04-08

**Authors:** Mariana Jordão França, Graciliano José França, Luciana Akemi Takahashi, Solena Ziemer Kusma Fidalski, Eduarda Casagrande Zanoni

**Affiliations:** 1 Universidade Positivo – UP, Curitiba, PR, Brasil.; 2 Universidade Federal do Paraná – UFPR, Hospital de Clínicas, Curitiba, PR, Brasil.

**Keywords:** Doppler ultrasound, brachial artery, anatomical variation

## Abstract

**Background:**

Anatomical variations in arteries of the upper limb, such as presence of an accessory brachial artery, are common and widely described in the literature, mainly in cadaveric studies, but it is now possible to diagnose them using vascular Doppler ultrasound.

**Objectives:**

To identify the incidence of accessory brachial artery using vascular Doppler ultrasound and compare the findings with cadaveric studies.

**Methods:**

This was a prospective study that examined 500 upper limbs of 250 volunteers assessed with vascular Doppler ultrasound using the Sonosite Titan portable ultrasound machine.

**Results:**

15.6% of the participants in our study had the accessory brachial artery anatomical variation. Our percentage is in line with the average rates found in cadaveric studies, which ranged from 0.2% to 22%. Being aware of this variation is fundamental in procedures such as peripheral venipuncture, arteriovenous fistula creation, catheterization, forearm flaps, emergency surgeries on the limb and even correction of fractures by cast.

**Conclusions:**

The accessory brachial artery is a frequent variant in the upper limb. The percentage of individuals with an accessory brachial artery in our study was 15.6%, which agrees with data from the literature on cadaveric studies.

## INTRODUCTION

The arterial circulation of the upper limbs starts with the subclavian artery, which is a branch of the brachiocephalic trunk on the right and a branch of the aortic arch on the left. After passing the lateral margin of the first rib, the subclavian artery is known as the axillary artery. At the inferior margin of the teres major muscle, the axillary artery becomes the brachial artery, which divides into two terminal branches, the radial and ulnar arteries, at the cubital fossa.^1^ This pattern of the arterial anatomy of the upper limb can vary at several different levels, from the axillary artery, proximal to the origin of the median nerve, to the superficial and deep palmar arches.^2^ Embryologically, the blood supply to the upper limb is provided by two arterial systems: one is via the primitive axillary artery, and the other is via the superficial arterial system. Intersegmental dorsal arteries that arise from the aorta and the capillary network of the mesenchyme form a primary axillary artery that becomes the brachial artery in the arm and the common interosseous artery in the forearm. This, in turn, gives rise to the anterior and posterior interosseous arteries.^3^ As the upper limb grows, the common interosseous artery is incorporated into the ulnar artery. Normally, the deep trunks maintain hemodynamic dominance, resulting in regression of the superficial system and its preanastomotic segments. Embryological studies propose that arterial anatomic variations of the upper limb can occur because of problems with angiogenesis and vasculogenesis.^4^ These embryological aspects mean that anatomic variants can occur frequently in the arteries of the upper limbs. One such variant is the accessory brachial artery, which is defined as coexistence of two brachial arteries that join back together before branching into the arteries of the forearm.

The accessory brachial artery may originate from the axillary artery or from the principal brachial artery itself. As soon as it crosses the elbow joint, it is renamed according to its course: radial, ulnar, superficial ulnar, interosseous, ulnoradial, or median.^5^ An accessory brachial artery was reported for the first time in 1830 by Green.^6^ The first anatomist to use this name in large cadaveric studies was Rodriguez-Niedenführ. Being aware of this arterial anatomic variant is essential in many areas of medicine.^7^

Usually, the majority of studies of anatomic variants of upper limb arteries are conducted with cadavers, which enables more detailed analysis of the course of variants.^7^ However, in view of their clinical importance, vascular Doppler ultrasound can be used as a diagnostic method.

Even allowing for the distortions in terms of detail and statistics in cadaveric and ultrasound studies, vascular Doppler ultrasound has proven to be an effective method for detection of this anatomic variant. Moreover, it is a noninvasive, widely available, and low-cost diagnostic method.

## OBJECTIVES

This study assessed the left and right brachial arteries of 250 volunteers using vascular Doppler ultrasound, with the objective of investigating the presence of the accessory brachial artery anatomic variant and comparing the data found in our study with the percentages reported in cadaveric studies.

## METHODS

This is a prospective, observational, cross-sectional, non-interventional study conducted in 250 volunteers by vascular Doppler ultrasound examination with a Sonosite Titan ultrasound machine and a linear transducer. The study participants were volunteers, enrolling in the sample spontaneously after the study had been advertised by the researchers. The sample size calculation was based on the confidence interval of a proportion. This type of calculation determines the prevalence of a characteristic in the population. The confidence level was 95%, and the margin of error was 5%. The estimated proportion in the population was 22%. The calculation indicated a sample of 264 people. The study achieved 95% of the estimated sample size.

Examinations were conducted of the left and right upper limbs, totaling 500 limbs examined. From an ultrasonographic point of view, presence or absence of the accessory brachial artery was determined using vascular Doppler ultrasound in the area close to the cubital fossa. When the artery was present, its continuation in the forearm was not evaluated. Variables selected for analysis were single or double brachial artery in the arm, and age and sex of volunteers. Vascular Doppler ultrasound was conducted with B-mode, color mode, and spectral analysis. Data on the volunteers were collected at a university in the city of Curitiba, Paraná, Brazil, in October and November of 2022. Data were computed sequentially by date and order of examination. Information was tabulated in an Excel spreadsheet, which was then used for statistical analysis with RStudio version 1.4 1103. The chi-square test and odds ratios were used. P values less than 0.05 were considered significant. The project was verified with the Strengthening the Reporting of Observational Studies in Epidemiology (STROBE) checklist for cross-sectional studies. All patients signed a free and informed consent form. The study was approved by the Ethics Committee on September 17, 2022, with Ethics Appraisal Submission Certificate number 62902122.1.0000.0093 and consolidated opinion number 5.649.858.

Inclusion criteria were voluntary participants of both sexes aged 18 to 60 years. Participants were excluded if it was only possible to conduct the examination on one upper limb, in order to avoid inputting unilateral data.

### Results

**Figure 1:**
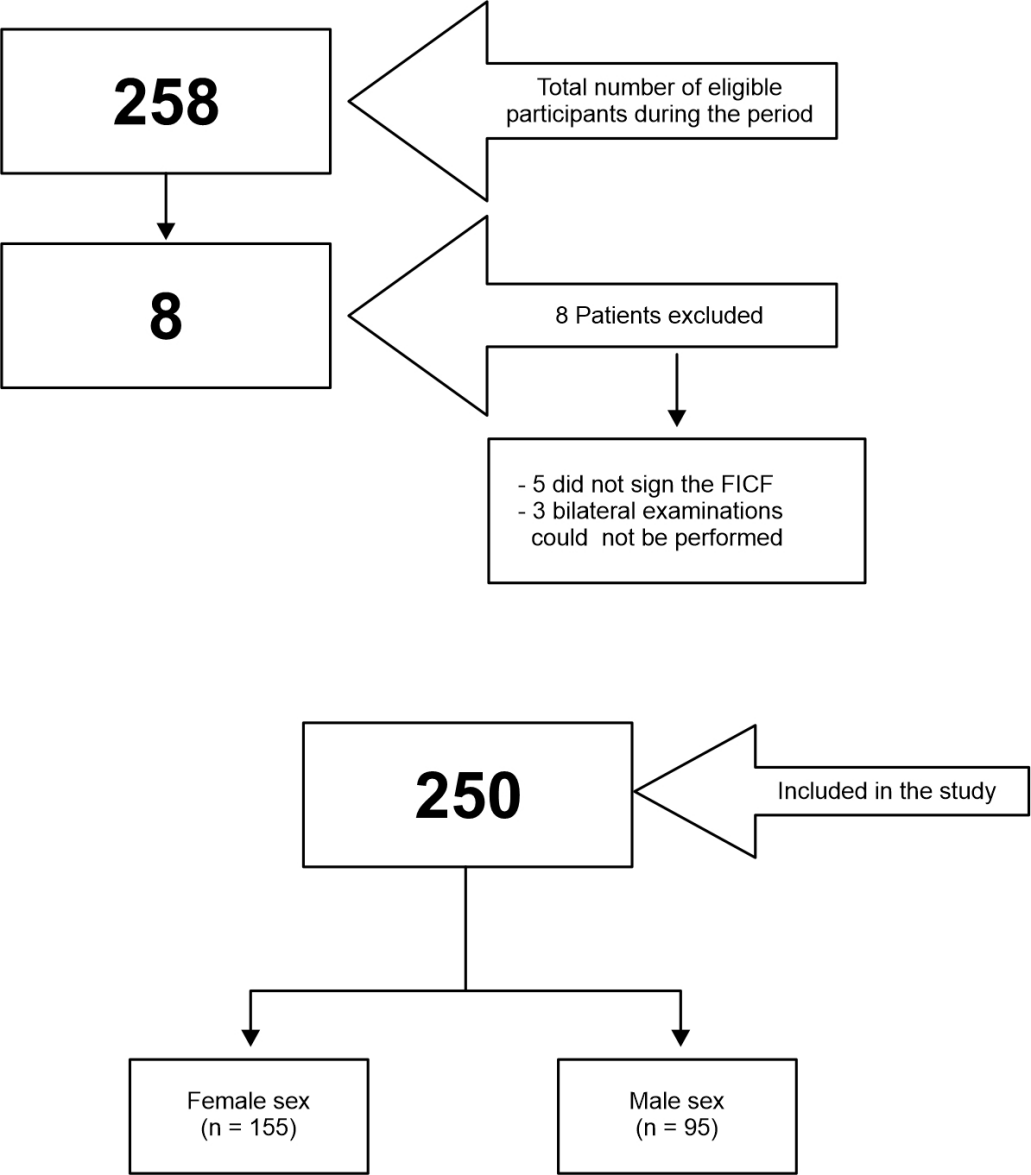
Flow diagram illustrating inclusion of patients in the study. FICF = free and informed consent form.

**Figure 2:**
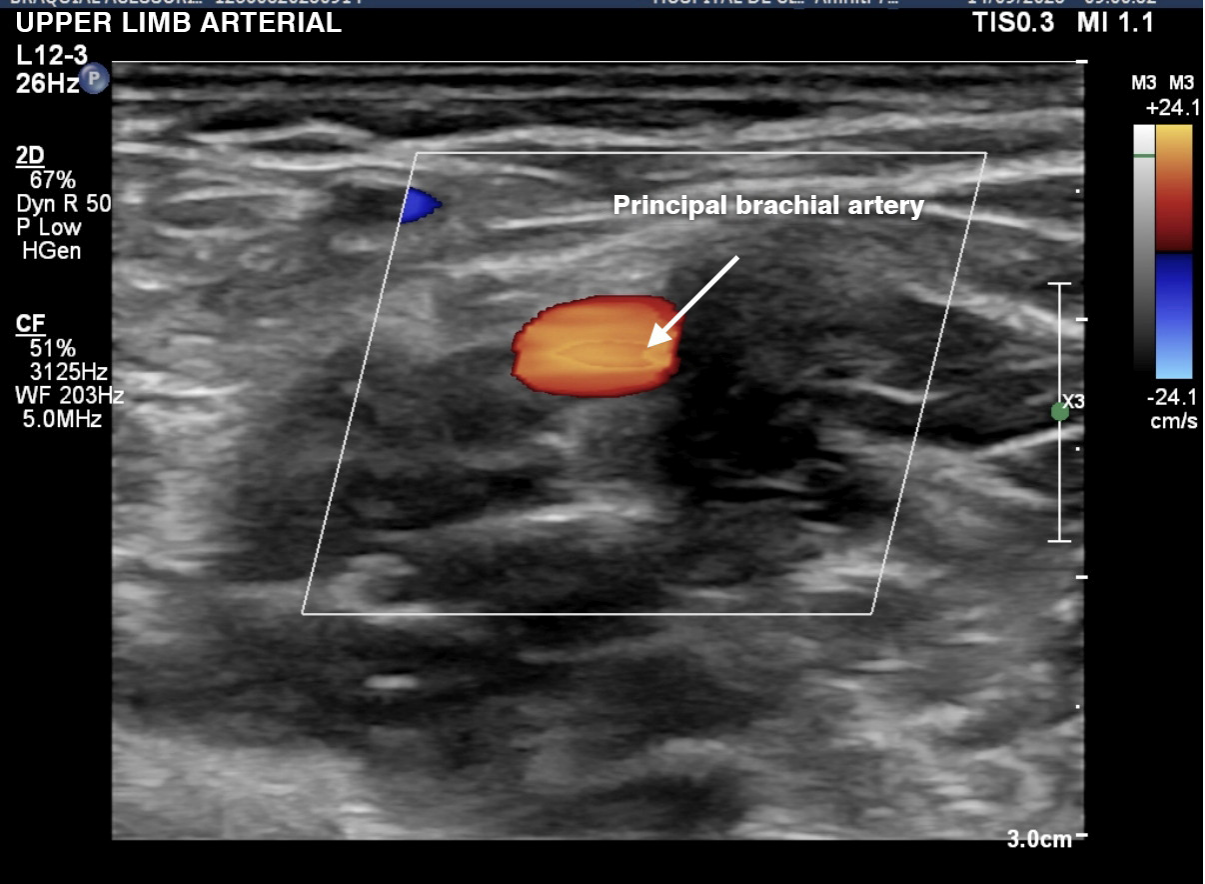
Doppler ultrasound in transverse view showing just the principal brachial artery, indicating that there is no anatomic variant of the artery.

**Figure 3:**
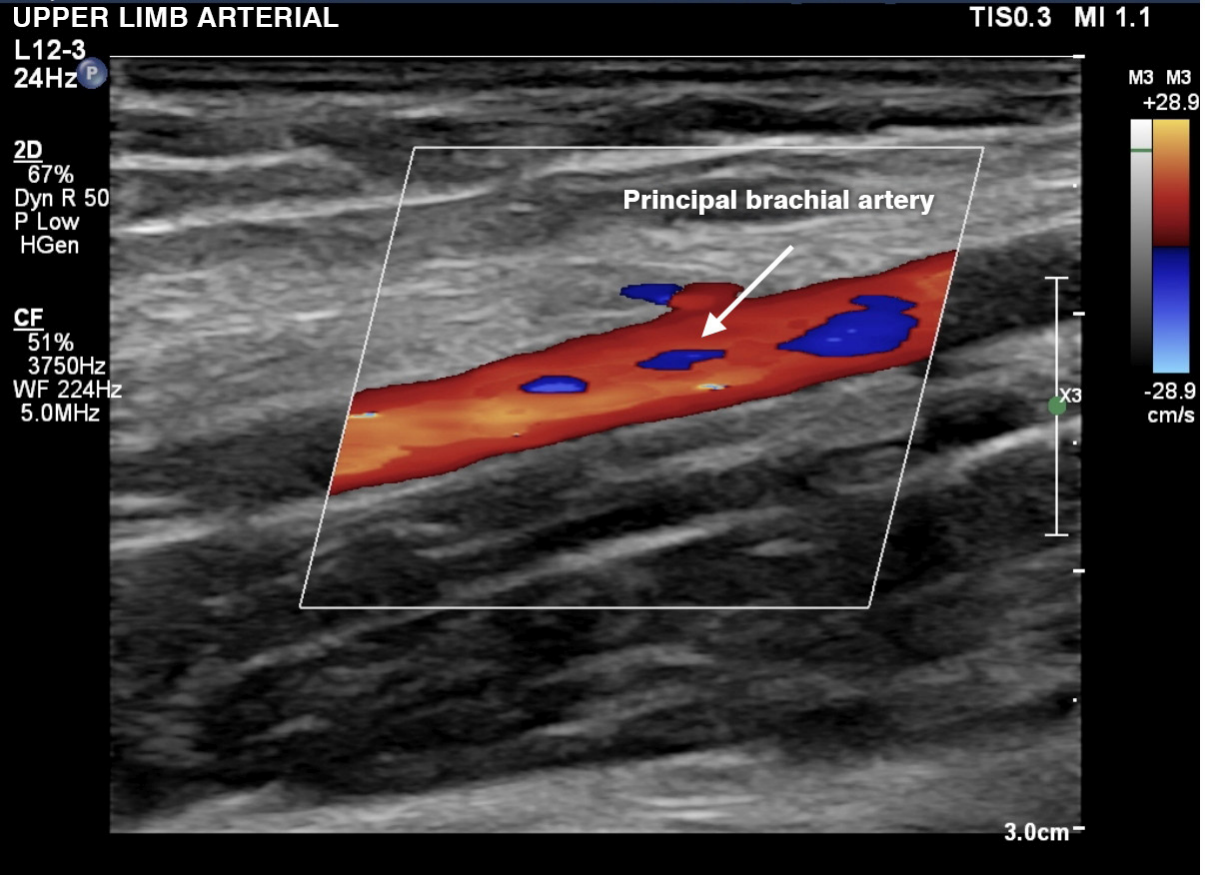
Doppler ultrasound in longitudinal view, showing the principal brachial artery

**Figure 4:**
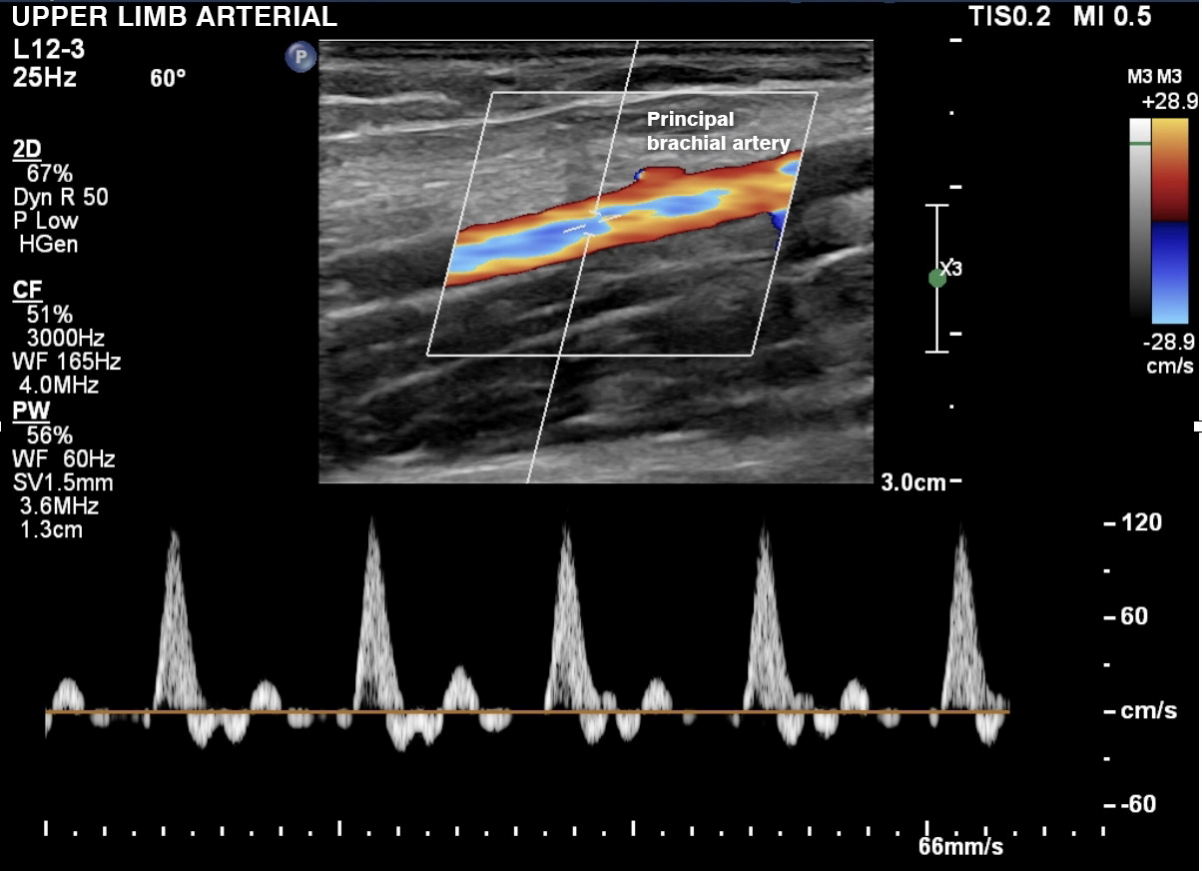
Doppler ultrasound in spectral mode, longitudinal view, showing normal flow in the brachial artery.

**Figure 5:**
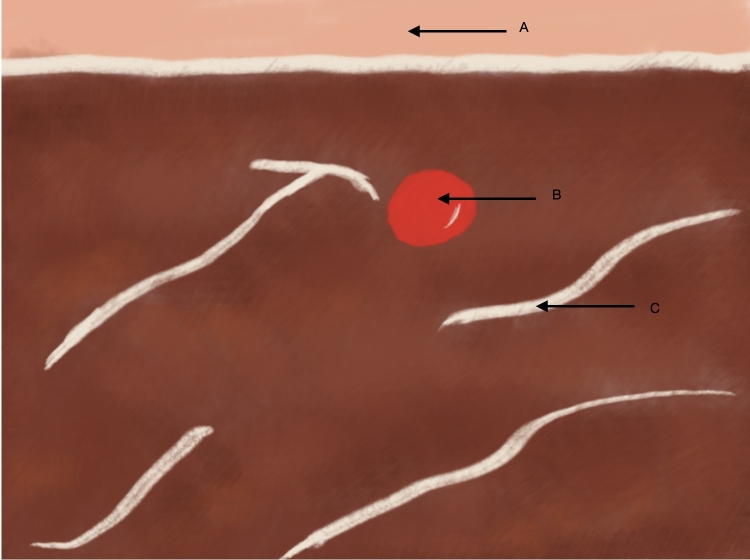
Schematic diagram illustrating Doppler ultrasound in transverse view. (A) indicates the skin and subcutis, (B) the principal brachial artery, and (C) the anterior muscles of the arm.

**Figure 6:**
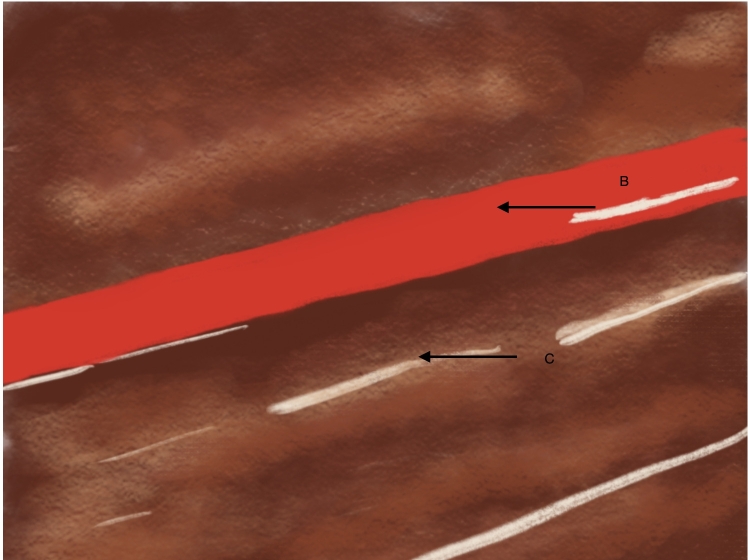
Schematic diagram illustrating Doppler ultrasound in longitudinal view. (B) indicates the principal brachial artery and (C) the anterior muscles of the arm.

**Figure 7:**
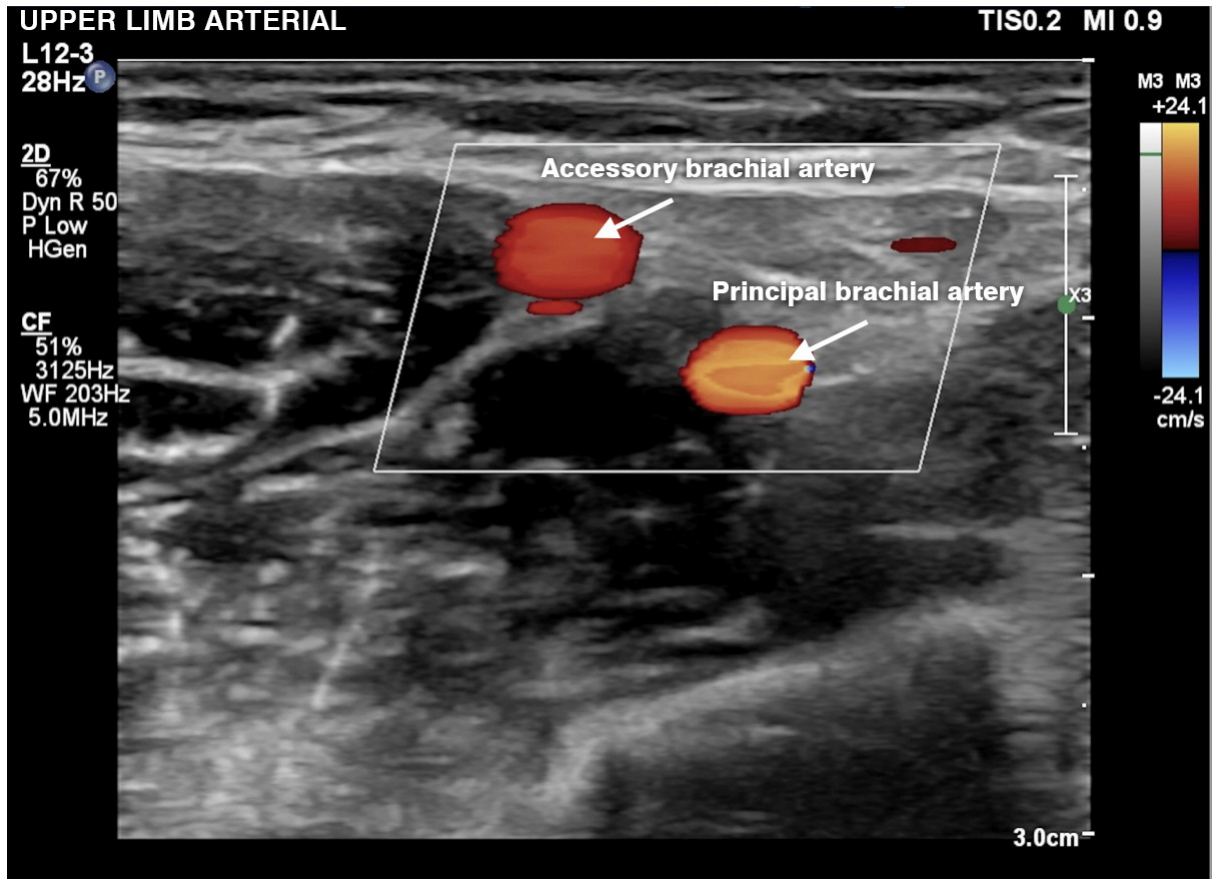
Doppler ultrasound in transverse view showing an accessory brachial artery and the principal brachial artery, indicating that there is an anatomic variant of the artery.

**Figure 8:**
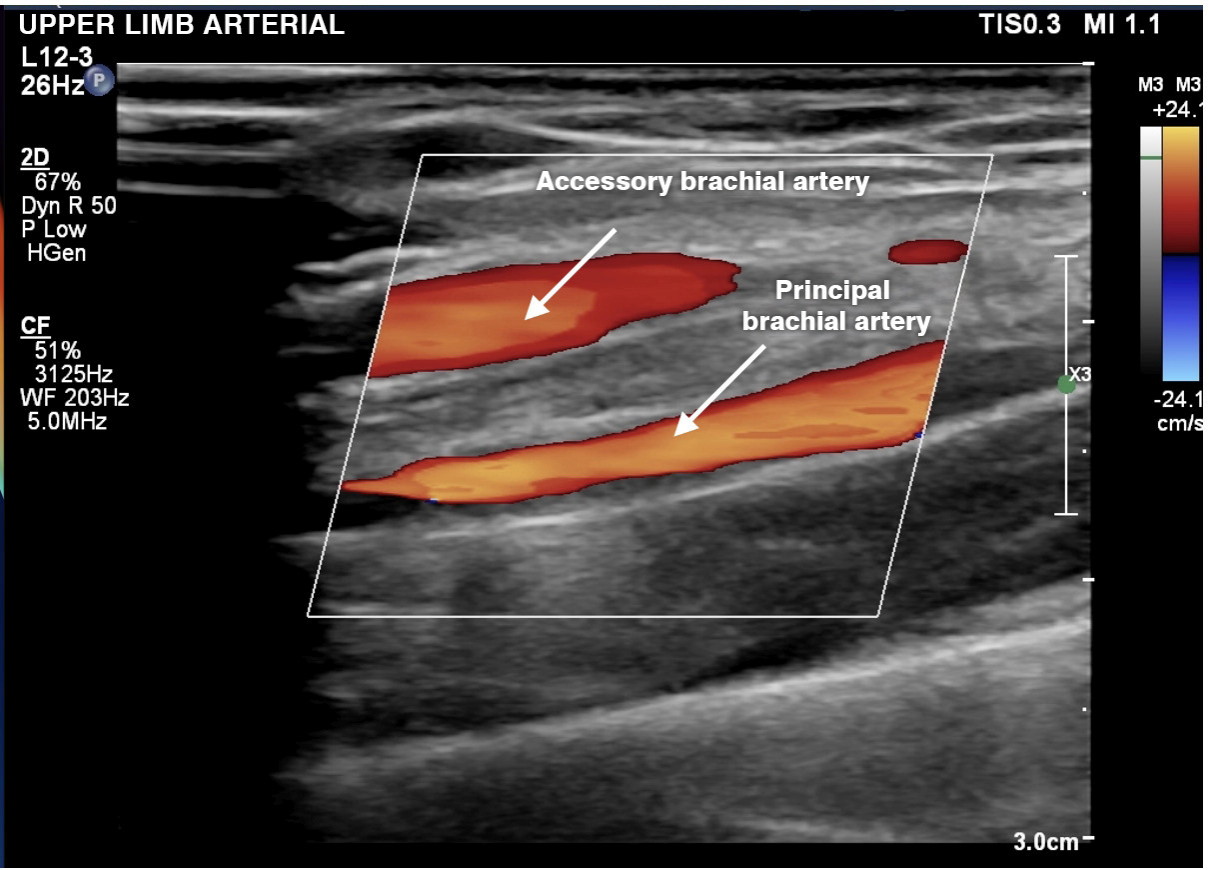
Doppler ultrasound in longitudinal view, showing an accessory brachial artery and the principal brachial artery.

**Figure 9:**
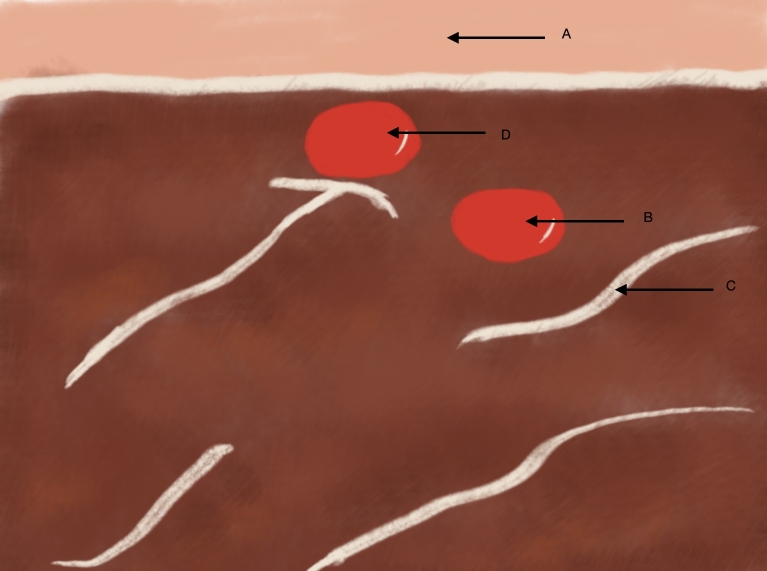
Schematic diagram illustrating Doppler ultrasound in transverse view. (A) indicates the skin and subcutis, (B) the principal brachial artery, (C) the anterior muscles of the arm, and (D) the accessory brachial artery

**Figure 10:**
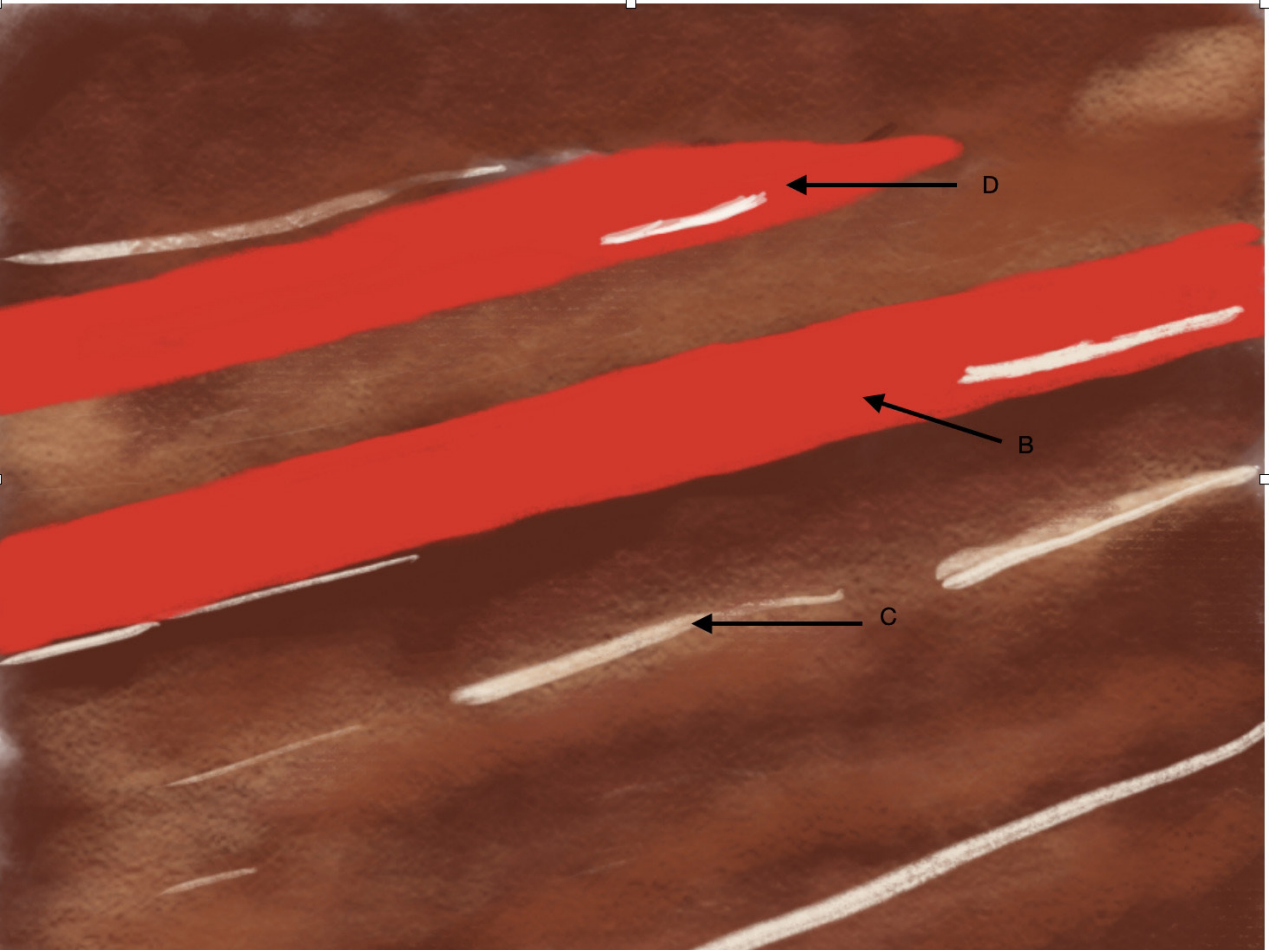
Schematic diagram illustrating Doppler ultrasound in longitudinal view. (B) indicates the principal brachial artery, (C) the anterior muscles of the arm, and (D) the accessory brachial artery.

A total of 258 volunteers were considered eligible. Eight people were excluded according to the exclusion criteria defined above (Figure 1). A total of 250 volunteers were included who had bilateral examinations with a portable ultrasound machine in October and November of 2022. The data obtained on the 500 upper limbs assessed showed that 39 limbs exhibited presence of an accessory brachial artery (15.6% of participants). The variables studied were brachial artery with no anatomic variant (Figures 2, 3, 4, 5 and 6) and brachial artery with an anatomic variant (Figures 7, 8, 9 and 10). Cases with an accessory brachial artery present were recorded as bilateral, right upper limb only, or left upper limb only (Table 1). The variant was more common in the right upper limb (20 volunteers) than the left upper limb (13 volunteers). A total of 6 volunteers exhibited the variation bilaterally. Once data had been collected, a statistical analysis was conducted. The chi-square test (Table 2) identified no significant difference between females and males in presence of an accessory brachial artery, since the p value was 0.40 with 95% reliability.

**Table 1:** Characteristics of study participants (n = 250).

Characteristic	n	%
**Age group (years)**		
18-24	195	78%
25-30	22	8.8%
31-40	16	6.4%
41-50	11	4.4%
51-60	6	2.4%
**Sex**		
Female	155	62%
Male	95	38%
**Brachial artery**		
No anatomic variant	211	84.4%
Anatomic variant	39	15.6%
Bilateral accessory brachial artery	6	2.4%
Accessory brachial artery in right upper limb	20	8%
Accessory brachial artery in left upper limb	13	5.2%

Source: The authors (2023).

**Table 2:** Inferential analysis of the association between anatomic variants of the brachial artery and sex.

Sex	Anatomic variant of the brachial artery (%)	No anatomic variant of the brachial artery (%)	Odds ratio (95%CI)	P
Female	27 (17.4%)	128 (82.6%)	1.46 (0.70-3.04)	0.40^1^
Male	12 (12.6%)	83 (87.4%)	1	

1 Chi-square test; 95%CI = 95% confidence interval. Source: The authors (2023).

## DISCUSSION

According to Arey, variations in the upper limb arteries can occur because of incomplete development, fusion of arteries that are usually separate, or persistence of vessels that are normally obliterated, but remain patent.^8^ Jurjus et al.^3^ report that one hypothesis for emergence of an accessory brachial artery is persistence of more than one intersegmental branch of the cervical artery, which should regress, but may persist and can sometimes even increase in size. Elnaiem et al.^7^ describe another possible origin of an accessory brachial artery as when normal regression of the proximal part of the radial artery does not occur. The same authors also state that another possible cause of this abnormality is abnormal branching and reunification with the axillary artery, giving rise to two arteries: the principal brachial artery and an accessory brachial artery.

The name given to the accessory brachial artery in the forearm depends on its course, and it can be termed brachioradial, brachioulnar, superficial brachioulnar, brachio-interosseous, brachio-ulnoradial, or even brachio-median,^8^ the last of which follows the course of the median nerve.^9^ Trifurcation of the brachial artery is also possible, as reported by Jacomo et al.,^10^ giving rise to radial, ulnar, and collateral superficial ulnar arteries.^10^ According to Devisankar et al.,^11^ the connection between the accessory brachial artery and the arteries of the forearm can be called an aberrant vessel. According to Elnaiem et al.,^7^ in the majority of cases the accessory brachial artery continues in the forearm as a collateral superficial ulnar artery, which contributes to formation of the superficial and deep palmar arches. Yoshiyuki et al.^12^ reported a case in which the accessory brachial artery followed a superficial course over the median nerve, while the principal brachial artery followed a path below the anterior flexor muscles of the arm and descended below the median nerve. Chakravarthi et al.^13^ assessed 70 limbs in a cadaveric study and concluded that the path of the accessory brachial artery (found in 11.43% of the sample) was more superficial and medial when compared to the deeper and more lateral course of the principal brachial artery.

The scientific literature was searched to compare the rates of accessory brachial artery observed in cadaveric studies with the findings of our study. Studies of the accessory brachial artery were identified with publication dates ranging from 1844, by Quain,^14^ to 2020, by Konarik.^15^ The lowest percentage was 0.2%, in the 1844 study by Quain.^14^ The highest percentage found was 22%, in a study published by Lippert and Pabst^16^ in 1985. The cadaveric studies found in the literature search also concluded that the accessory brachial artery variant is more frequent in the right upper limb.^17^ Our study examined 250 subjects with vascular Doppler ultrasound, finding that 39 individuals had an accessory brachial artery, equating to 15.6% of the sample. In the majority of cases (20 patients), the variant was in the right upper limb. The percentage observed in our study is within the average found in cadaveric studies, which varied from 0.2 to 22%, and which also concluded that the vessel is more prevalent in the right upper limb.^13^

Knowledge of anatomical variation of the brachial artery bifurcation is essential, since it constitutes one of the most important variations of the upper limb arteries and is very relevant to medical practice.^18^ Its superficial course makes the accessory brachial artery very vulnerable to traumas. In the Pan African Medical Journal, a case was reported of accessory brachial artery injury after posterior elbow joint dislocation caused by a fall.^19^ Use of casts to correct forearm fractures can also injure and compress an accessory brachial artery.^19^ Accidental intra-arterial administration of injections, such as during preparation for angiography, can cause thrombosis of an accessory brachial artery, or even gangrene of the limb, which can lead to amputation of fingers or the forearm.^20^ When performing venipuncture to draw peripheral blood, an accessory brachial artery can be mistaken for the median vein in the cubital fossa because of its superficial course. Accidental puncture can cause pseudoaneurysm formation. It is important to know about the possibility of an accessory brachial artery variant before brachioradial procedures such as catheterization. The accessory brachial artery is not suitable for transradial catheterization procedures because of its smaller caliber.^21^ A study at the Charles University in Prague concluded that the accessory brachial artery is two or even three times smaller than the principal brachial artery, which is insufficient to allow the catheter to pass.^9,22^ In 1998, Nunoo-Mensah reported a case in which, hand ischemia occurred after transradial catheterization for coronary bypass surgery because of injury to the brachial artery in a patient with absent ulnar artery.^23^ According to Kachlik et al.,^22^ the accessory brachial artery is more frequently hypoplastic or stenotic, because of its smaller caliber. Surgical procedures that require a forearm flap involve a high possibility of injury to the accessory brachial artery during the procedure.^21^ On the other hand, there are also positives to having an accessory brachial artery. According to Chen et al.,^20^ the artery can be an alternative option for construction of an arteriovenous fistula for hemodialysis. Moreover, it can also contribute to collateral circulation in the event of occlusion of the principal brachial artery.^10,24^

The main limitation of this study is the fact that the authors only examined the bifurcation of the brachial artery and did not investigate its continuation and branches in the forearm. It was not therefore possible to classify the course of the accessory brachial artery, when present, as brachioradial, brachioulnar, superficial brachioulnar, brachio-interosseous, brachio-ulnoradial, or even brachio-median.

Since the course and branching pattern of this artery vary between different cases in the literature, the ideal would be to understand the anatomy and variations of course as a whole. In order for medical professionals to be aware of the consequences of these variants, knowledge of the accessory brachial artery is essential and is extremely important in many medical fields, such as radiology, orthopedics, oncology, nephrology, intensive medicine, and even surgical areas that involve trauma and reconstruction.

It can be concluded that the accessory brachial artery is a variant that is frequently found in the upper limb. The percentage of individuals with an accessory brachial artery in our study was 15.6%, which agrees with cadaveric studies found in the scientific literature.
