# Supplementary material for: Assessment of anatomic variations of the brachial artery bifurcation using vascular Doppler ultrasound: a cross-sectional study
Source: J Vasc Bras. 2024 Apr 8;23:e20230117. doi: 10.1590/1677-5449.202301172 (PMC11042541; doi:10.1590/1677-5449.202301172)
Supplement: Translation

# Translation: Avaliação das alterações anatômicas da bifurcação da artéria braquial através do eco-Doppler vascular: um estudo transversal

**DOI:** 10.1590/1677-5449.202301171

**Authors:** Mariana Jordão França, Graciliano José França, Luciana Akemi Takahashi, Solena Ziemer Kusma Fidalski, Eduarda Casagrande Zanoni

**Affiliations:** 1 Universidade Positivo – UP, Curitiba, PR, Brasil.; 2 Universidade Federal do Paraná – UFPR, Hospital de Clínicas, Curitiba, PR, Brasil.

**Keywords:** ultrassom Doppler, artéria braquial, variação anatômica

## Abstract

**Contexto:**

Variações anatômicas em artérias do membro superior, como a presença da artéria braquial acessória, são comuns e amplamente descritas na literatura, principalmente por estudos em cadáveres. No entanto, atualmente, é possível realizar o diagnóstico através do eco-Doppler vascular.

**Objetivos:**

Identificar a incidência da artéria braquial acessória pelo eco-Doppler e comparar os achados com estudos cadavéricos.

**Métodos:**

Tratou-se de um estudo prospectivo em 500 membros superiores de 250 voluntários avaliados pelo eco-Doppler com o aparelho portátil de ultrassom Sonosite Titan.

**Resultados:**

Dos participantes do nosso estudo, 15,6% apresentaram a variação anatômica da artéria braquial acessória. A porcentagem está dentro da média encontrada em estudos cadavéricos, que varia de 0,2 até 22%. Ter conhecimento dessa variação é fundamental em procedimentos como punção venosa periférica, fístula arteriovenosa, cateterismo, retalhos de antebraço, cirurgias de emergência no membro superior e até mesmo correção de fraturas por gesso.

**Conclusões:**

A artéria braquial acessória é uma variante frequente no membro superior. O percentual de indivíduos com a artéria braquial acessória em nosso estudo foi de 15,6% e coincide com os dados da literatura de estudos cadavéricos.

## INTRODUÇÃO

A circulação arterial dos membros superiores inicia com a artéria subclávia, que é ramo do tronco braquiocefálico à direita e ramo do arco aórtico à esquerda. Após a margem lateral da primeira costela, a artéria subclávia passa a ser denominada artéria axilar. Na borda inferior do músculo redondo maior, a artéria axilar passa a se chamar artéria braquial, a qual divide-se em seus dois ramos terminais, artérias radial e ulnar - na fossa cubital^1^ . Este padrão arterial do membro superior pode variar em diferentes níveis, desde a artéria axilar, proximal à origem do nervo mediano, até o nível dos arcos palmares superficial e profundo^2^ . Embriologicamente, o suprimento sanguíneo do membro superior ocorre por dois sistemas arteriais: um é através da artéria axilar primitiva, e o outro, pelo sistema arterial superficial. Artérias intersegmentares dorsais que emergem da aorta e da rede de capilares do mesênquima formam uma artéria axilar primária que se torna artéria braquial no braço e artéria interóssea comum no antebraço. Esta, por sua vez, dá origem às artérias interósseas anterior e posterior^3^ . Conforme o membro superior cresce, a artéria interóssea comum acaba por se incorporar à artéria ulnar. Normalmente, os troncos profundos mantêm uma dominância hemodinâmica, resultando na regressão do sistema superficial e dos seus segmentos pré-anastomóticos. Estudos embriológicos propõem que variações anatômicas arteriais do membro superior podem ocorrer por problemas na angiogênese e vasculogênese^4^ . Devido a estes aspectos embriológicos, variações anatômicas podem ocorrer frequentemente nas artérias do membro superior. Uma delas é a artéria braquial acessória, a qual é definida como a coexistência de duas artérias braquiais que se reúnem antes de se ramificar nas artérias do antebraço.

Essa artéria braquial acessória pode se originar da artéria axilar ou da própria braquial principal. Assim que essa cruza a articulação do cotovelo, é renomeada de acordo com o seu curso: radial, ulnar, ulnar superficial, interóssea, ulnoradial ou mediana^5^ . A artéria braquial acessória foi reportada pela primeira vez em 1830 por Green^6^ . O primeiro anatomista a aplicar essa nômina em estudos abrangentes cadavéricos foi Rodriguez-Niedenführ. Estar ciente de tal variação anatômica arterial é fundamental em diversas áreas médicas^7^ .

Usualmente, a maioria dos estudos de variação anatômica de artérias do membro superior são realizados em cadáveres, o que permite maior detalhamento do percurso^7^ . Todavia, devido a sua importância clínica, pode ser utilizado o eco-Doppler vascular como método diagnóstico.

Mesmo com distorções a respeito do detalhamento e das estatísticas nos estudos cadavéricos e ultrassônicos, o eco-Doppler vascular demonstrou ser uma prática eficaz na detecção da variação anatômica. Além disso, esse método diagnóstico é rápido, não invasivo, amplamente disponível e de baixo custo.

## OBJETIVOS

O estudo avaliou as artérias braquiais direita e esquerda de 250 voluntários através do eco-Doppler vascular, com o intuito de investigar a presença da variação anatômica da artéria braquial acessória e comparar os dados encontrados em nosso estudo com a porcentagem relatada em estudos cadavéricos.

## MÉTODOS

Tratou-se de um estudo prospectivo, observacional, transversal e não intervencionista através da realização de exames de eco-Doppler vascular com aparelho de ultrassom Sonosite Titan, transdutor linear, em 250 voluntários. Os participantes da pesquisa eram voluntários, contribuindo para a amostra de forma espontânea após a divulgação do estudo pelos pesquisadores.. Assim, o cálculo do tamanho da amostra foi realizado por meio do intervalo de confiança de uma proporção. Esse cálculo determina a prevalência de uma característica da população. O nível de confiança foi de 95%, e a margem de erro foi de 5%. A proporção estimada na população foi de 22%. O cálculo apontou uma amostra de 264 pessoas. Nesta pesquisa, atingiu-se 95% da amostra prevista.

Os exames foram realizados nos membros superiores direito e esquerdo, perfazendo 500 membros avaliados. Do ponto de vista ultrassônico, a presença ou ausência da artéria braquial acessória foi estudada por meio da realização do eco-Doppler vascular na região próxima à fossa cubital. Não foi avaliada a continuação dessa artéria, quando presente, no antebraço. As variáveis selecionadas foram artéria braquial única ou dupla no braço, sexo e idade dos voluntários. O eco-Doppler vascular foi realizado através do modo B, modo colorido e análise espectral. Foram colhidos dados de voluntários em uma universidade na cidade de Curitiba, no Paraná, entre outubro e novembro de 2022. Os dados foram computados sequencialmente por data e ordem da realização do exame nos participantes. As informações foram tabuladas em planilha no Excel, com as quais foi feita uma análise estatística pelo programa RStudio versão 1.4 1103. Os testes utilizados foram o qui-quadrado e a razão de chances. Valores de p menor que 0,05 foram considerados significativos. O projeto passou pela *checklist* do Strengthening the Reporting of Observational Studies in Epidemiology (STROBE) para estudos seccionais. Todos os pacientes assinaram o termo de consentimento livre e esclarecido. O estudo foi aprovado pelo Comitê de Ética em 17 de setembro de 2022, com o número de Certificado de Apresentação de Apreciação Ética 62902122.1.0000.0093 e parecer consubstanciado 5.649.858.

Os critérios de inclusão foram participantes de ambos os sexos, entre 18 e 60 anos, seguindo o critério de voluntariedade. Foram excluídos participantes dos quais só foi possível a realização do exame de um único membro superior, a fim de evitar a entrada de dados unilaterais.

## RESULTADOS

Um total de 258 voluntários foram considerados elegíveis. Oito pessoas foram excluídas conforme os critérios de exclusão previamente definidos (Figura 1). Foram incluídos 250 voluntários que tiveram seus exames bilaterais realizados entre outubro e novembro de 2022 através do ultrassom portátil. Dos dados obtidos através de 500 membros superiores avaliados, 39 membros apresentaram a presença da artéria braquial acessória (15,6% dos participantes). As variáveis estudadas foram artéria braquial sem variação anatômica (Figuras 2, 3, 4, 5 e 6) e artéria braquial com variação anatômica (Figuras 7, 8, 9 e 10). No caso da presença da artéria braquial acessória, foi detalhado se a variação ocorreu bilateralmente, no membro superior direito ou no membro superior esquerdo (Tabela 1). A presença da variação foi mais comum no membro superior direito (20 voluntários) do que no membro superior esquerdo (13 voluntários).No total, 6 voluntários apresentaram a variação em ambos os braços. Com os dados coletados, foi realizada uma análise estatística. Com a aplicação do teste do qui-quadrado (Tabela 2), foi possível afirmar que não existiu diferença significativa entre o sexo feminino e masculino para a presença da artéria braquial acessória, já́ que o valor de p encontrado foi de 0,40 e a razão de confiabilidade foi de 95%.

**Figura 1:** Fluxograma dos pacientes incluídos no estudo. TCLE = termo de consentimento livre e esclarecido.

**Figura 2:** Eco-Doppler em corte transversal demonstrando a presença de apenas a artéria braquial principal, indicando que não há variação anatômica dessa artéria.

**Figura 3:** Eco-Doppler em corte longitudinal demonstrando a artéria braquial principal.

**Figura 4:** Eco-Doppler em modo espectral, corte longitudinal, demonstrando fluxo normal na artéria braquial.

**Figura 5:** Desenho esquemático demonstrando eco-Doppler no corte transversal. Em (A) estão sinalizados pele e subcutâneo, em (B) artéria braquial principal e em (C) músculos anteriores do braço.

**Figura 6:** Desenho esquemático demonstrando eco-Doppler no corte longitudinal. Em (B) está sinalizada a artéria braquial principal e em (C) músculos anteriores do braço.

**Figura 7:** Eco-Doppler em corte transversal demonstrando a presença da artéria braquial acessória e da artéria braquial principal, indicando que há variação anatômica dessa artéria.

**Figura 8:** Eco-Doppler em corte longitudinal demonstrando a artéria braquial acessória e a artéria braquial principal.

**Figura 9:** Desenho esquemático demonstrando eco-Doppler no corte transversal. Em (A) estão sinalizados pele e subcutâneo, em (B) artéria braquial principal, em (C) músculos anteriores do braço e em (D) a artéria braquial acessória.

**Figura 10:** Desenho esquemático demonstrando eco-Doppler no corte longitudinal. Em (B) está sinalizada a artéria braquial principal, em (C) músculos anteriores do braço e em (D) a artéria braquial acessória.

**Tabela 1:** Descrição das características dos participantes da pesquisa (n = 250).

Característica	n	%
**Faixa etária (anos)**		
18-24	195	78%
25-30	22	8,8%
31-40	16	6,4%
41-50	11	4,4%
51-60	6	2,4%
**Sexo**		
Feminino	155	62%
Masculino	95	38%
**Artéria Braquial**		
Sem variação anatômica	211	84,4%
Com variação anatômica	39	15,6%
Artéria braquial acessória bilateral	6	2,4%
Artéria braquial acessória no membro superior direito	20	8%
Artéria braquial acessória no membro superior esquerdo	13	5,2%

**Fonte:** Autores (2023).

**Tabela 2:** Análise inferencial da associação entre variação anatômica na artéria braquial por sexo.

Sexo	Variação anatômica na artéria Braquial (%)	Sem variação anatômica na artéria braquial (%)	Razão de chances (IC95%)	Valor de p
Feminino	27 (17,4%)	128 (82,6%)	1,46 (0,70-3,04)	0,40^1^
Masculino	12 (12,6%)	83 (87,4%)	1	

1 Teste do qui-quadrado; IC95% = intervalo de confiança de 95%. **Fonte:** Autores (2023).

## DISCUSSÃO

De acordo com Arey, variações nas artérias do membro superior podem ocorrer devido a desenvolvimento incompleto, pela fusão de artérias usualmente separadas ou por vasos que seriam normalmente obliterados, mas que persistem pérvios^8^ . Jurjus et al.^3^ relatam que uma das hipóteses do surgimento da artéria braquial acessória é a persistência de mais de um ramo intersegmentar da artéria cervical, que deveria regredir, mas persiste e pode eventualmente aumentar de tamanho. Já Elnaiem et al.^7^ relatam a possibilidade de a artéria braquial acessória ter origem quando não ocorre a regressão usual da parte proximal da artéria radial. Esses autores ainda afirmam que outra possibilidade para essa anormalidade é a ramificação anormal e a reunificação com a artéria axilar, dando origem a duas artérias: a braquial principal e a braquial acessória.

A denominação da artéria braquial acessória no antebraço depende de sua continuação, podendo ser denominada de braquiorradial, braquioulnar, braquioulnar superficial, braquio-interóssea, braquio-ulnoradial ou, até mesmo, braquio-mediana^8,^ sendo que nessa última a artéria acompanha o trajeto do nervo mediano^9^ . Pode existir também uma trifurcação da artéria braquial reportada por Jacomo et al.^10^ , a qual origina as artérias radial, ulnar e ulnar colateral superficial. Segundo Devisankar et al.^11^ , essa conexão entre a artéria braquial acessória com as artérias do antebraço pode ser denominada de vaso aberrante. Segundo Elnaiem et al.^7^ , na maioria das vezes a artéria braquial acessória continua no antebraço como uma artéria ulnar superficial colateral, o que contribui para a formação dos arcos palmares superficiais e profundos. Yoshiyuki et al.^12^ reportam um caso em que a artéria braquial acessória possui trajeto superficial e acima do nervo mediano, enquanto a artéria braquial principal segue um trajeto abaixo dos músculos flexores anteriores do braço e desce abaixo do nervo mediano. Chakravarthi et al.^13^ concluíram em seu estudo cadavérico, após 70 membros avaliados, que o trajeto da artéria braquial acessória (encontrada em 11,43% de sua amostra) se demonstrou mais superficial e medial quando comparado a um percurso mais profundo e lateralizado da artéria braquial principal.

Foi realizada pesquisa na literatura científica para comparar a presença da artéria braquial acessória em estudos cadavéricos com a nossa pesquisa. Foram encontrados estudos a respeito da artéria braquial acessória, com datas de publicação que variam desde 1844, por Quain^14^ , até 2020, por Konarik et al.^15^ . A menor porcentagem encontrada foi de 0,2%, em um estudo feito por Quain em 1844^14^ . Já a maior porcentagem encontrada foi de 22%, em um estudo realizado por Lippert e Pabst^16^ em 1985. Estudos cadavéricos da literatura utilizada na pesquisa também concluíram que a variação da artéria braquial acessória está presente com mais frequência no membro superior direito^17^ . Nosso estudo, tendo examinado 250 indivíduos, constatou, através do eco-Doppler vascular, que houve a presença de 39 indivíduos com a artéria braquial acessória, totalizando 15,6% da amostra. Na maioria dos casos (20 pacientes), a variação surgiu no membro superior direito. A porcentagem do nosso estudo está dentro da média encontrada em estudos cadavéricos, que variou de 0,2 a 22%, e que também concluiu que a maior prevalência desse vaso é no membro superior direito^13^ .

O conhecimento das variações anatômicas da bifurcação da artéria braquial é fundamental, já que corresponde a uma das principais variações das artérias dos membros superiores e é muito relevante na prática médica^18^ . Por causa de seu trajeto superficial, a artéria braquial acessória está muito vulnerável a traumas. Na literatura do Jornal de Medicina Pan Africano, foi relatada a lesão da artéria braquial acessória após deslocamento da articulação posterior do cotovelo, resultante de uma queda em escada^19^ . A utilização de gesso para correção de fraturas no antebraço também pode lesar e comprimir a artéria braquial acessória^19^ . A aplicação acidental de injeção intra-arterial, como em preparos para angiografia, pode causar trombose na artéria braquial acessória, ou até mesmo gangrena do membro, o que pode levar a amputação dos dedos ou do antebraço^20^ . Em casos de punção venosa para coleta de sangue periférico, a artéria braquial acessória pode ser confundida com a veia mediana na fossa cubital devido a seu trajeto superficial. Sua punção acidental pode levar a formação de um pseudoaneurisma. É relevante ter conhecimento sobre a possível variação da artéria braquial acessória em braquiorradial em procedimentos como cateterismo. A artéria braquial acessória não suporta procedimentos de cateterismo transradial por ser menos calibrosa^21^ . Um estudo da Universidade Carolina, em Praga, concluiu que a artéria braquial acessória é duas ou até três vezes menor que a artéria braquial principal, o que não permite a passagem do cateter^9,22^ . Nunoo-Mensah reportou em 1998 um caso em que, após cateterismo transradial para *by-pass* coronário em paciente com ausência da artéria ulnar, ocorreu isquemia da mão devido a lesão da artéria braquial^23^ . Segundo Kachlik et al.^22^ , devido ao seu menor calibre, a artéria braquial acessória é, com maior frequência, hipoplásica ou estenótica. Procedimentos cirúrgicos que utilizam retalhos antebraquiais radiais têm alta possibilidade de lesar a artéria braquial acessória no procedimento^21^ . Por outro lado, existem pontos positivos em ter a presença da artéria braquial acessória. Segundo Chen et al.^20^ , a artéria pode servir como uma alternativa para realização de uma fístula arteriovenosa para hemodiálise. Além disso, também pode contribuir com uma circulação colateral em caso de oclusão da artéria braquial principal^10,24^ .

A principal limitação deste estudo é o fato de que os autores examinaram apenas a bifurcação da artéria braquial, não sendo avaliada a sua continuidade e ramos no antebraço. Não foi possível definir o trajeto da artéria braquial acessória, quando presente, em braquiorradial, braquioulnar, braquioulnar superficial, braquio-interóssea, braquioulnoradial ou, até mesmo, braquio-mediana.

Como o curso e o padrão de ramificação dessa artéria variam entre casos na literatura, o ideal é compreender a anatomia e a variação do trajeto como um todo. A fim de alertar os profissionais médicos a respeito das consequências dessa variação, ter conhecimento a respeito da artéria braquial acessória é essencial e de extrema importância em diversas práticas médicas como radiologia, ortopedia, oncologia, nefrologia, medicina intensiva e até mesmo áreas cirúrgicas que envolvam operações de trauma e reconstrução.

É possível concluir que a artéria braquial acessória é uma variante encontrada com frequência no membro superior. O percentual de indivíduos com a artéria braquial acessória em nosso estudo foi de 15,6%, dado que coincide com estudos cadavéricos encontrados na literatura científica.
